# Multivariate hierarchical frameworks for modeling delayed reporting in count data

**DOI:** 10.1111/biom.13188

**Published:** 2019-11-29

**Authors:** Oliver Stoner, Theo Economou

**Affiliations:** ^1^ Department of Mathematics University of Exeter Exeter UK

**Keywords:** Bayesian methods, censoring, generalized Dirichlet, multivariate count data, notification delay, underreporting

## Abstract

In many fields and applications, count data can be subject to delayed reporting. This is where the total count, such as the number of disease cases contracted in a given week, may not be immediately available, instead arriving in parts over time. For short‐term decision making, the statistical challenge lies in predicting the total count based on any observed partial counts, along with a robust quantification of uncertainty. We discuss previous approaches to modeling delayed reporting and present a multivariate hierarchical framework where the count generating process and delay mechanism are modeled simultaneously in a flexible way. This framework can also be easily adapted to allow for the presence of underreporting in the final observed count. To illustrate our approach and to compare it with existing frameworks, we present a case study of reported dengue fever cases in Rio de Janeiro. Based on both within‐sample and out‐of‐sample posterior predictive model checking and arguments of interpretability, adaptability, and computational efficiency, we discuss the relative merits of different approaches.

## 1 INTRODUCTION

In many biostatistical applications where count data are collected, a situation can arise where the available reported count is believed to be less than or equal to the true count. Delayed reporting in particular is where the total observable count, which may still be less than the true count, will only be available after a certain amount of time. In some situations, information will trickle in over time so that the current total count gets ever closer to the true count, before eventually reaching the final total observable count.

**Figure 1:**
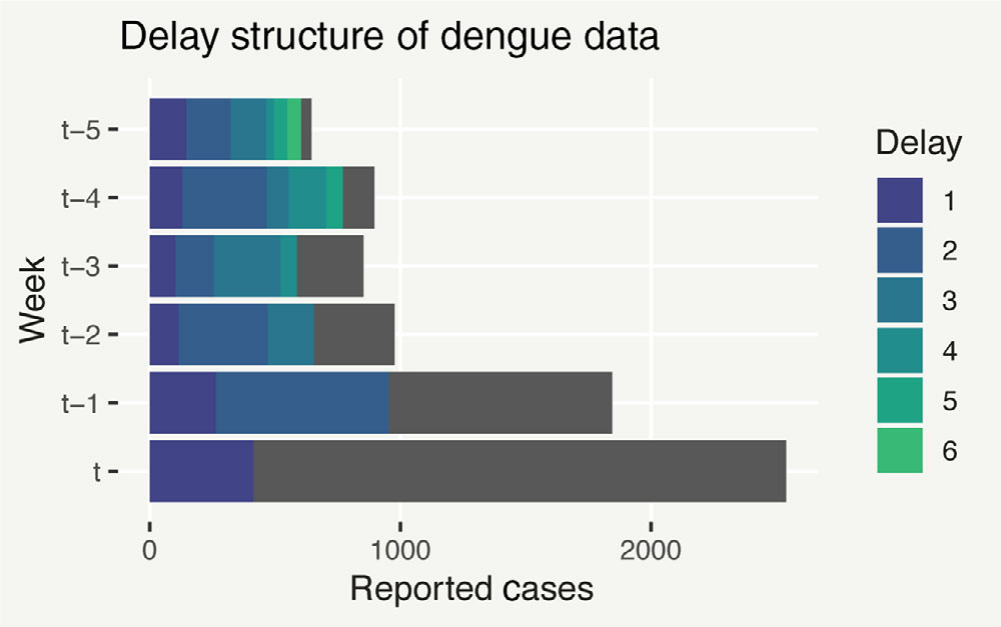
Bar plot of Rio de Janeiro dengue cases in the weeks leading up to time t=114. The gray bars represent the total (as yet unobserved) number of reported cases, while the different colored bars show the number of cases reported after each week of delay. This figure appears in color in the electronic version of this article, and any mention of color refers to that version

An example of this situation is the occurrence of dengue fever, a viral infection spread by mosquitoes, in Rio de Janeiro. Delayed reporting implies that, at the end of some week *t*, we will have only observed a portion of the total observable number of cases $y_{t}$ which were contracted over the course of week *t*. At t+1, a further portion will become available and so on, such that after a number of weeks $y_{t}$ eventually becomes known. Figure 1 shows an instance of the data, where t=114. The gray portions of each bar represent the yet unknown cases as of week *t*. For week t−1, we only have 2 weeks worth of information because we only have data that arrived in weeks t−1 and *t*. Likewise, for week t−2 we only have 3 weeks worth of information and so on.

Reporting delay is a problem when decisions based on the total count need to be made before it has been completely observed. Figure 1, for example, illustrates that it can take months before $y_{t}$ is known. This impedes the response time to severe outbreaks and puts lives at risk. It is therefore necessary to make predictions about the current state of the disease based on the partial counts observed (nowcasting), to enable action such as issuing of warnings for predicted epidemics before they have been completely detected by the data. This motivates a statistical treatment of delayed reporting, aiming to predict the total count based on corresponding available partial counts. Further goals include predicting total counts which have not occurred yet (forecasting) and learning about the structure of the delay mechanism, to inform improvements in reporting.

In this article, we review and evaluate previous statistical approaches to modeling delayed reporting of counts (Section 2). We then propose a general framework for modeling count data with discrete‐time delays, which is sufficiently flexible to be used for a range of applications (Section 3). We present two variations of this framework which differ in how the expected delay mechanism is modeled. In Section 4, we present a case study of dengue fever counts in Rio de Janeiro to test the efficacy of the proposed framework compared to existing approaches. Here, also in a more comprehensive prediction experiment presented in Web Appendix A, we base model assessment on posterior predictive checking of nowcasting and forecasting performance. In Section 5, we discuss underreporting in the final observed count and how the proposed framework can be adapted to account for it. Finally, Section 6 concludes with a discussion of interpretability, adaptability, and ease of implementation.

## 2 BACKGROUND

We begin by introducing some notation. Let $y_{t}$ be the total observable count occurring at temporal unit t∈T. We refer to $y_{t}$ as “observable” because this may still be an underrepresentation of the true count $x_{t}\geq y_{t}$, an issue we return to in Section 5. Suppose that after some (temporal) delay unit (eg, 1 week) a portion of $y_{t}$, $z_{t,1}\leq y_{t}$, has been reported. At the next delay unit, we observe an additional portion of $y_{t}$, denoted as $z_{t,2}$. This continues so that at each delay unit d∈{1,⋯,D} (where *D* is the maximum possible delay) we observe a count $z_{t,d}$ and $\sum_{j=1}^{d}z_{t,j}$ gets closer to $y_{t}$.

The biostatistical literature on modeling delayed reporting is well established, notably for correcting AIDS or HIV records (eg, Rosinska *et al*. (2018)). Historically, the task of correcting the delayed reporting has been separated from the task of modeling or forecasting the incidence of the total count (see for instance Brookmeyer and Damiano, 1989, and Harris, 1990). However, this ignores the joint uncertainty in the incidence of the total count and the presence of delay. For example, suppose that at time *t* the number of cases reported in the first week $z_{t,1}$ is unusually low. This could either be because a low proportion of $y_{t}$ was reported in the first week, or because $y_{t}$ was itself unusually low, or both. Differentiating between these cases is vital for reliable prediction, so from this point on, we only focus on approaches which jointly model the delay mechanism and the total count.

### 2.1 Multinomial mixture approach

A sensible approach for modeling delayed reporting involves the idea of jointly modeling $z_{t,d}|y_{t}$ at the same time as the totals $y_{t}$. Höhle and an der Heiden (2014) propose modeling the delayed counts as $z_{t}|y_{t}∼\text{multinomial}\left(p_{t},y_{t}\right)$. Here $p_{t,d}$ is the expected proportion of $y_{t}$ which will be reported at delay *d* and is modeled as arising from the generalized Dirichlet(α,β) (GD) distribution (Wong, 1998), an extension of the Dirichlet(α) distribution (Kotz *et al*., 2004). If $p=\left(p_{1},\cdots ,p_{k}\right)∼\text{Dirichlet}\left(\alpha \right)$, then for $\phi =\sum_{i=1}^{k}\alpha_{i}$, $E\left[p_{i}\right]=\mu_{i}=\alpha_{i}/\phi$, $\mathrm{Var}\left[p_{i}\right]=\mu_{i}\left(1-\mu_{i}\right)/\left(\phi +1\right)$ and $\text{Cov}\left[p_{i},p_{j}\right]=-\mu_{i}\mu_{j}/\left(\phi +1\right)$, so the covariance of any pair is negative. The conditional distributions are $p_{1}∼\text{Beta}\left(\alpha_{1},\Sigma_{j=2}^{k}\alpha_{j}\right)$; $q_{i}∼\text{Beta}\left(\alpha_{i},\Sigma_{j=i+1}^{k}\alpha_{j}\right)$, where $p_{i}=q_{i}\left(1-\sum_{j=1}^{i-1}p_{j}\right)$ and finally $p_{k}=1-\sum_{j=1}^{k-1}p_{j}$. The GD introduces a free parameter $\beta_{i}$ so that each $q_{i}∼\text{Beta}\left(\alpha_{i},\beta_{i}\right)$. The increased number of parameters (2k−1 compared to *k* in the Dirichlet) results in a more general covariance structure, for example, allowing for positive covariances (Wong, 1998). As such it is a very flexible distribution for use in modeling multivariate proportion or count data, where the different elements have distinct variances or indeed an unusual covariance structure (eg, Stoner *et al*., 2019b). In Höhle and an der Heiden (2014), α and β are temporally constant and $y_{t}$ is a latent Poisson variable:

(1) $$\begin{array}{c} y_{t}|\lambda_{t}∼\text{Poisson}\left(\lambda_{t}\right);\;\log\left(\lambda_{t}\right)=f\left(t\right), \end{array}$$

where f(t) represents a combination of covariate or random effects. Wang *et al*. (2018) also apply this approach to monitoring of food‐borne diseases.

The assumption that α and β are time‐invariant can be viewed as a restriction in capturing any delay mechanism which varies systematically over time, potentially inhibiting nowcasting and forecasting precision. Höhle and an der Heiden (2014) address this by replacing the GD component with a more conventional multinomial regression. The modeled quantity is then $\nu_{t,d}$, the expected proportion of counts which will be reported at delay *d* out of those which are yet‐to‐be‐reported:

(2) $$\log\left(\frac{\nu_{t,d}}{1-\nu_{t,d}}\right)=g\left(t,d\right);\;p_{t,d}=\nu_{t,d}\left(1-\sum_{i=1}^{d-1}p_{t,i}\right),\;$$

where g(t,d) is a linear combination of covariate effects. Quantity $\nu_{t,d}$ is termed the “hazard” as it is akin to a hazard function in survival regression. This model allows for temporal heterogeneity in the delay mechanism; however, it is in part more restrictive. Note that this is in essence a multivariate generalization of the binomial framework for underreporting presented in Stoner *et al*. (2019a), where $y_{t}$ is made up of only two partial counts: an observable total count and an unobservable remainder that was missed due to underreporting.

Removing the GD variability risks confounding variability in the delay mechanism with variability in the total count $y_{t}$ when nowcasting. We illustrate this by considering the predictive distribution for unobserved totals $y_{t}$ given partial counts $z_{t}$: $p\left(y_{t}|z_{t}\right)\propto p\left(z_{t}|y_{t}\right)p\left(y_{t}\right)$. Here $p(z_{t}|y_{t})$ is multinomial, which lacks flexibility in the variance since the means, variances, and covariances are all defined wholly by $p_{t}$. If there is excess variability (overdispersion) in $z_{t}|y_{t}$, this is likely to be erroneously absorbed by $p(y_{t})$. For example, if $z_{t,1}/y_{t}$ is too high for the multinomial to reasonably capture given $p_{t,1}$, then predictions of $y_{t}$ may be too high when nowcasting. Moreover, if both the mean and correlation structure in $z_{t,s}|y_{t,s}$ are exclusively defined by $p_{t,s}$, then flexibility in capturing unusual covariance structures is limited.

### 2.2 Conditional independence approach

A similar approach presented in Salmon *et al*. (2015) extends the Poisson model for $y_{t}$ to a negative‐binomial (NB), where the additional parameter θ allows for overdispersion:

(3) $$\begin{array}{ccc} y_{t}|\lambda_{t},\theta & ∼ & \text{NB}\left(\lambda_{t},\theta \right);\;\log\left(p_{t,d}\right)=g\left(t,d\right), \end{array}$$

where $\lambda_{t}$ is modeled as in (1). Here the multinomial probabilities $p_{t,d}$ are modeled directly with a log‐link. The marginal distribution for $z_{t}$ is then also NB:

(4) $$z_{t,d}|p_{t,d},\lambda_{t}∼\text{NB}\left(\mu_{t,d}=p_{t,d}\lambda_{t},\theta \right);$$

(5) $$\log\left(\mu_{t,d}\right)=\log\left(p_{t,d}\lambda_{t}\right)=f\left(t\right)+g\left(t,d\right).$$

The resulting marginal model is effectively (conditional on dispersion parameters) a NB generalized linear model (GLM) (Dobson and Barnett, 2018) for $z_{t,d}$. It is also possible to arrive at this model by generalizing the Chain‐Ladder method (Mack, 1993), often used in the field of actuarial statistics for projecting ultimate losses from delayed insurance claims.

The advantage of only considering the marginal model is that it can be easily implemented in a variety of likelihood frameworks (such as generalized additive models; Wood, 2017), as well as Bayesian ones. For example, Bastos *et al*. (2019) use integrated nested Laplacian approximations (INLA) (Lindgren and Rue, 2015) to apply this framework to dengue fever in Rio de Janeiro and to spatiotemporal Severe Acute Respiratory Infection (SARI) data in the state of Paraná (Brazil). However, there is an inherent danger in directly modeling $z_{t}$: when the multinomial model is not able to capture all of the variability in the delay mechanism, the dispersion parameter θ must account for this, in addition to any overdispersion in $y_{t}$. This amalgamation of overdispersion from both $y_{t}$ and $z_{t,d}$ means that estimates for θ may lead to excessive variance in any predicted $y_{t}$ when simulating from (3). We illustrate this using simulated data in Section 1 of Web Appendix A.

Instead, we may predict $y_{t}$ as $y_{t}=\sum_{d=1}^{D}z_{t,d}$. This has two issues: First, uncertainty in the delay component of $z_{t,d}$ is potentially transferred to $y_{t}$ through the summation. This may result in predictive uncertainty (eg, as quantified by 95% prediction intervals) that is prohibitively large, particularly when forecasting into the future where no $z_{t,d}$ are available. Second, we would want $\text{Var}\left[y_{t}\right]=\text{Var}\left[\sum_{d=1}^{D}z_{t,d}\right]=\sum_{i=1}^{D}\sum_{j=1}^{D}\text{Cov}\left[z_{t,i},z_{t,j}\right]$ to be captured well. In turn, $\text{Cov}[z_{t,i},z_{t,j}]$ must be captured well, but this is restricted by the assumption that $z_{t,d}$ are independent (given $\mu_{t,d}$). In particular, this ignores a considerable source of positive covariance in $z_{t}$. Consider that (3) is equivalent to a Poisson‐gamma mixture, that is, $y_{t}|\gamma_{t}∼\text{Poisson}\left(\gamma_{t}\right)$, where $\gamma_{t}∼\text{Gamma}\left(\theta ,\theta \lambda_{t}^{-1}\right)$. The marginal model for $z_{t,d}$ is therefore $\text{Poisson}(p_{t,d}\gamma_{t})$, where $E\left[z_{t,d}|\gamma_{t}\right]=p_{t,d}\gamma_{t}$, such that $\gamma_{t}$ induces positive covariance in $z_{t}$.

In the following section, we present a general modeling framework, which can capture heterogeneity in the delay mechanism and can appropriately separate variability and uncertainty in the delay mechanism from the model of the total count.

## 3 GENERALIZED DIRICHLET‐MULTINOMIAL FRAMEWORK

We begin by defining a NB model for the total counts:

(6) $$\begin{array}{c} y_{t}|\lambda_{t},\theta ∼\text{NB}\left(\lambda_{t},\theta \right);\;\log\left(\lambda_{t}\right)=f\left(t\right), \end{array}$$

with f(t) a general function as in Section 2. Given $y_{t}$, the model for the partial counts is

(7) $$\begin{array}{ccc} z_{t}|p_{t},y_{t} & ∼ & \text{Multinomial}(p_{t},y_{t}). \end{array}$$

As discussed in Section 2.1, assuming that $p_{t}$ are fixed given any random effects or covariates is problematic: there is a risk of confounding variability in the delay mechanism with variability in $y_{t}$ when nowcasting, and there is limited flexibility in capturing unusual covariance structures. Both of these issues can be addressed by assuming $p_{t}∼\text{GD}\left(\alpha_{t},\beta_{t}\right)$, where

(8) $$\begin{array}{ccc} & & p(p_{1},p_{2},\cdots ,p_{k}|\alpha ,\beta )\\ & & \;=p_{k}^{\beta_{k-1}-1}\prod_{i=1}^{k-1}\left[\frac{p_{i}^{\alpha_{i}-1}}{B(\alpha_{i},\beta_{i})}{\left(\sum_{j=i}^{k}p_{j}\right)}^{\beta_{i-1}-\left(\alpha_{i}+\beta_{i}\right)}\right]. \end{array}$$

The marginal distribution of $z_{t}$ is therefore a generalized Dirichlet‐multinomial$\left(\alpha_{t},\beta_{t},y_{t}\right)$ (GDM), with probability mass function:

(9) $$\begin{array}{ccc} & & p(z_{1},z_{2},\cdots ,z_{k}|\alpha ,\beta ,y)\\ & & \;=\frac{\Gamma (y+1)}{\Gamma (z_{k}+1)}\prod_{i=1}^{k-1}\left[\frac{\Gamma \left(z_{i}+\alpha_{i}\right)\Gamma \left(\sum_{j=i+1}^{k}z_{j}+\beta_{i}\right)}{B\left(\alpha_{i},\beta_{i}\right)\Gamma \left(z_{i}+1\right)\Gamma \left(\alpha_{i}+\beta_{i}+\sum_{j=i}^{k}z_{j}\right)}\right].\; \end{array}$$

For nowcasting. we need predictive inference for $y_{t}$ given any observed $z_{t,d}$ (as well as any preceding observed $y_{t}$). Using Markov chain Monte Carlo (MCMC; as is done here), this is possible by sampling the corresponding not‐yet‐observed $z_{t,d}$ and $y_{t}$. We therefore need to be able to sample from the conditional distributions $z_{t,d}|z_{t,1},\cdots ,z_{t,d-1},y_{t}$, which are given by

(10) $$z_{i}|z_{-i},\alpha ,\beta ,y∼\text{Beta-Binomial}\left(\alpha_{i},\beta_{i},n_{i}=y-\sum_{j<i}z_{j}\right);\;$$

(11) p(zi∣z−i,α,β,y)=niziB(zi+αi,ni−zi+βi)B(αi,βi).

To sensibly model structured variability in the delay mechanism, we re‐parametrize (10) in terms of mean $\nu_{t,d}$ and dispersion $\phi_{t,d}$, where $\alpha_{t,d}=\nu_{t,d}\phi_{t,d}$ and $\beta_{t,d}=\left(1-\nu_{t,d}\right)\phi_{t,d}$.

Having already observed some delayed counts $z_{t,1},\cdots ,z_{t,d-1}$ corresponding to the total count $y_{t}$, $\nu_{t,d}$ represents the proportion of the remaining (so far unreported) counts, we expect to be reported in the next delay step *d*. Variability about $\nu_{t,d}$ is controlled by $\phi_{t,d}$, which can be generally characterized as a function of time and delay:

(12) $$\log\left(\phi_{t,d}\right)=h\left(t,d\right).$$

Unlike the GLM approach, predictive inference for $y_{t}$ is based on both the delayed counts $z_{t}$ and previous observed values $y_{t^{\prime }}$, for $t^{\prime }\leq t-D+1$. Using MCMC automatically generates predictive samples from $y_{t}|z_{t},y_{t^{\prime }}$. Furthermore, when nowcasting or forecasting, uncertainty in the delay mechanism only propagates into predictive uncertainty for $y_{t}$ through the available partial counts (observed elements of $z_{t}$) for that week. Uncertainty in the behavior of any unobserved $z_{t}$ (or corresponding $\nu_{t}$) does not influence predictions of $y_{t}$. In the following subsections, we present two alternative models for the proportions $\nu_{t,d}$.

### 3.1 Hazard model

The first model for the delay mechanism is a natural extension of the multinomial regression in Höhle and an der Heiden (2014). The expected delay mechanism is characterized directly in terms of $\nu_{t,d}$, which is akin to a hazard function in survival regression:

(13) $$\begin{array}{c} \log\left(\frac{\nu_{t,d}}{1-\nu_{t,d}}\right)=g\left(t,d\right). \end{array}$$

**Figure 2:**
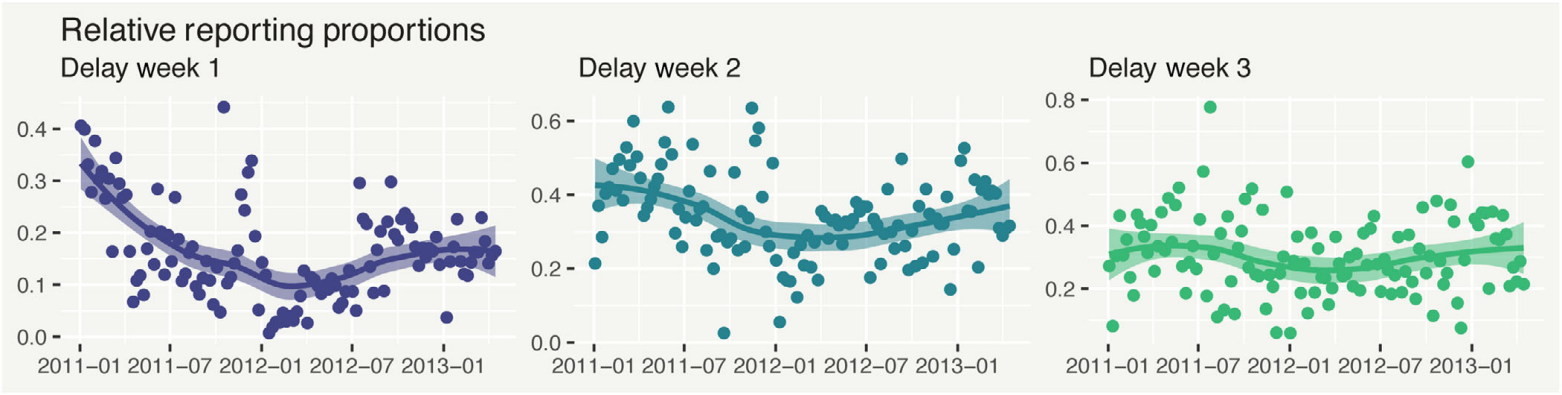
Proportion of not‐yet‐reported dengue cases ($y_{t}-\sum_{j=0}^{d-1}z_{t,j}$, with $z_{t,0}=0$), with super‐imposed LOESS estimates, reported in the same week they occurred (*d* = 1, left), in the week after they occurred (*d* = 2, center), and in week *d* = 3 (right). This figure appears in color in the electronic version of this article, and any mention of color refers to that version

The intuition is to think about how the temporal structure in the proportion of reported cases differs across delay levels. Figure 2 shows the proportion of dengue cases reported in each of the first three delay weeks, out of all those yet‐to‐be‐reported. In the left plot, the proportion of cases reported in the same week they occurred (*d* = 1) generally decreases over 2011 before increasing again. We could, therefore, define g(t,1) as a smooth function of time.

While this characterization is intuitive for the first delay, it loses interpretability as the delay increases. For example, it is difficult to intuitively understand the expected proportion reported after 6 weeks of delay, out of those unreported after 5 weeks. We could just include a different smooth function of time in each g(t,d), but it is not immediately obvious how to simplify this for less complicated temporal structures and reduce the risk of overparametrization. In the following subsection, we present an equally flexible but more interpretable delay model.

### 3.2 Survivor model

**Figure 3:**
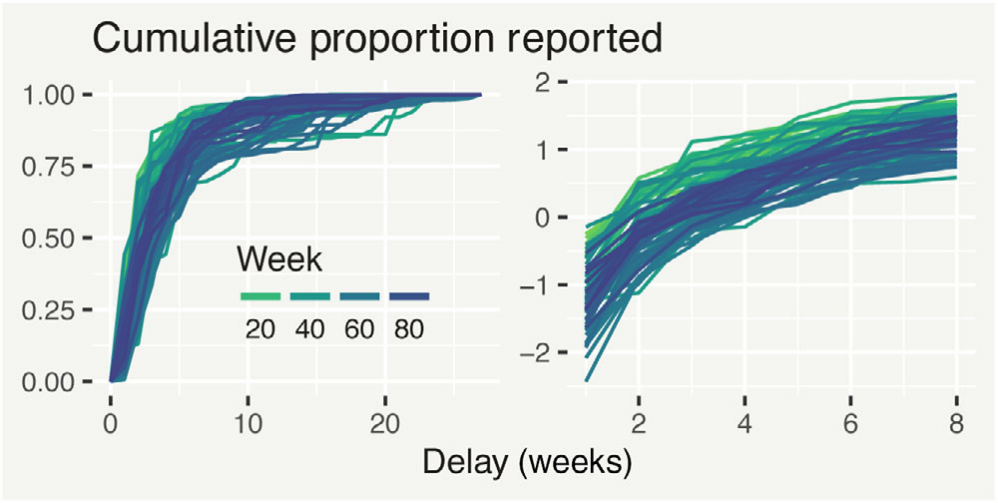
Cumulative proportion of total reported dengue cases reported after each week of delay, with no transformation (left) and a probit transformation (right). This figure appears in color in the electronic version of this article, and any mention of color refers to that version

Instead of different temporal structures for each delay, we can think about a delay structure for each time point. In particular, we can examine how the cumulative proportion of cases, defined as $s_{t,d}=\sum_{j=1}^{d}z_{t,j}/y_{t}\in \left[0,1\right]$, varies with time. The two plots in Figure 3 show $s_{t,d}$ and $\text{probit}(s_{t,d})$ plotted against *d* for dengue, where a clear pattern emerges: a collection of similar curves, shifted up and down as time varies. For example, curves around t=80 are usually lower down compared to earlier realizations (eg, around t=20). This motivates a general model for the expected cumulative proportions:

(14) $$\begin{array}{c} \text{probit}\left(E\left[s_{t,d}\right]\right)=\text{probit}\left(S_{t,d}\right)=g\left(t,d\right), \end{array}$$

where g(t,d) is once again a general combination of covariates or random effects. We refer to this as the “survivor” variant of the GDM framework, as $S_{t,d}$ is akin to a survivor function. The familiar relative proportions $\nu_{t,d}$ can be computed by

(15) $$\begin{array}{c} \nu_{t,d}=\frac{S_{t,d}-S_{t,d-1}}{1-S_{t,d-1}}. \end{array}$$

Including delay‐time interactions in g(t,d) results in equivalent flexibility to the hazard variant in capturing complex delay mechanisms. However, a key advantage of the survivor variant is that it remains intuitive for an arbitrary number of delay levels. Moreover, it can be easily reduced to more efficiently capture simple delay mechanisms (eg, as in Figure 3).

In the subsequent section, we will apply comparable GDM hazard, GDM survivor and GLM models to dengue fever data, discussing their relative merits with respect to performance in model checking, nowcasting, and forecasting.

## 4 CASE STUDY

Dengue fever is a mosquito‐borne viral infection that may evolve into a potentially fatal condition known as severe dengue (WHO, 2018). It is a major societal burden, particularly in Brazil which reports more dengue cases than any other country (Silva *et al*., 2016). Effective response to dengue requires early detection (WHO, 2018), so preparedness of healthcare providers for outbreaks relies on timely information. Though the reporting of dengue cases to the Brazilian national surveillance system (SINAN) is mandatory (Silva *et al*., 2016), it can take weeks/months of delay for the weekly number of reported cases to approach a final count. As such, statistical models are used to correct delays and predict outbreaks before the total count is available (Bastos *et al*., 2019).

Here we consider data on dengue cases in Rio de Janeiro, occurring in weeks t=1 (week commencing (w/c) January 3, 2011) to t=120 (w/c April 15, 2013). For illustration, we assume that present day, denoted by *t*_0_, is week $t_{0}=114$ (w/c March 4, 2013). Furthermore, we assume the total count to be the number of cases reported within 6 months of occurrence. This means that $y_{t}=\sum_{d=1}^{27}z_{t,d}$, where $z_{t,1}$ (d=1) represents the number of cases reported in the same week they occurred. Similarly, $z_{t,2}$ (d=2) represents the number of cases reported in the week after they occurred and so on. For $t_{0}=114$, we have 88 weeks of fully observed total counts $y_{t}$, while *y*_89_–*y*_114_ are partially observed and must be nowcasted. Unobserved $y_{t}$ for t>114 constitute the forecasting period.

**Figure 4:**
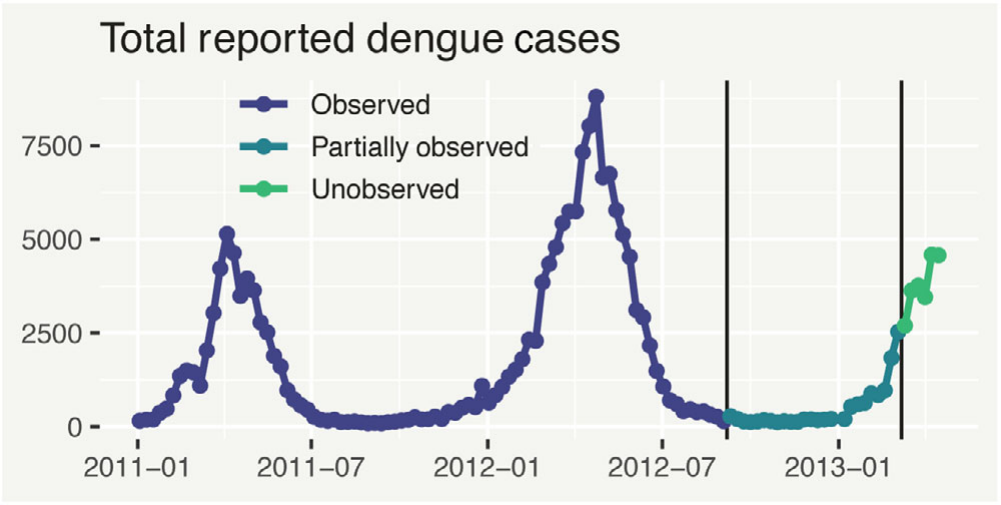
Total number of reported dengue cases from 2011 onwards in Rio de Janeiro. Different colors represent which data are fully observed, partially observed or unobserved at week t=114 (March 2013). This figure appears in color in the electronic version of this article, and any mention of color refers to that version

Figure 4 shows the associated time series of $y_{t}$. There is some evidence of seasonality, with outbreaks starting at the beginning of the calendar year, ending approximately 6 months later. This may be because dengue incidence is connected to the time of and climatological conditions (Morales *et al*., 2016). Some nonseasonal temporal structure is also evident, for example, the 2012 outbreak is more severe than the one in 2011. Finally, we can see (with hindsight) that at $t_{0}=114$ we are well into a third outbreak, with worse to come.

### 4.1 Formulation of competing GDM and GLM models

Modeling all available partial counts $z_{t,d}$ (for d=1,…,27) maximizes predictive information, albeit at a potentially high computational cost. In some cases, it may be more pragmatic to only model $z_{t,d}$ up to $d=D^{\prime }$, alongside the sum of the remaining counts $r_{t}=y_{t}-\sum_{d=1}^{D^{\prime }}z_{t,d}$. In the GDM approach, we achieve this by only including the conditional models for the first $D^{\prime }$ partial counts, such that the remainder $r_{t}$ is modeled implicitly, while in the GLM approach $r_{t}$ is modeled by (4), as if it were an individual $z_{t,d}$. In Section 4 of Web Appendix A, we present a sensitivity experiment which illustrates that, at least for these data, uncertainty in predictions of $y_{t}$ is unaffected for $t>t_{0}-D^{\prime }$. Choice of $D^{\prime }$ can therefore be viewed as a trade‐off between computation time (which increases linearly with $D^{\prime }$), and the number of weeks prior to *t*_0_ for which predictions must be as precise as possible. Here we choose $D^{\prime }=8$, which maximizes prediction precision for the last 8 weeks (including *t*_0_).

The model based on the GDM hazard framework is defined by

(16) $$y_{t}∼\text{NB}\left(\lambda_{t},\theta \right);\;\log\left(\lambda_{t}\right)=\iota +\alpha_{t}+\eta_{t};$$

(17) $$z_{t}|y_{t}∼\text{GDM}\left(\nu_{t},\phi ,y_{t}\right);\;\;\log\left(\frac{\nu_{t,d}}{1-\nu_{t,d}}\right)=\psi_{d}+\beta_{t,d},$$

where $\nu_{t}$ and ϕ are parameters of the beta‐binomial conditionals, as described in (10)–(12). In the GDM survivor model, the model for $\nu_{t,d}$ in (17) is replaced by $\text{probit}\left(S_{t,d}\right)=\psi_{d}+\beta_{t}$, where $\nu_{t}$ relates to $S_{t}$ as in (15). Finally, the model based on the GLM framework is

(18) $$z_{t,d}∼\text{NB}\left(\mu_{t,d},\theta \right);\;\log\left(\mu_{t,d}\right)=\iota +\alpha_{t}+\eta_{t}+\psi_{d}+\beta_{t,d}.\;$$

In all models, $\eta_{t}$ is a penalized cyclic cubic spline (Wood, 2017) defined over weeks 1,…,52, aimed at capturing within‐year temporal variation in the total dengue cases $y_{t}$. Similarly, $\alpha_{t}$ is a penalized cubic spline defined over the whole time range, aimed at capturing nonseasonal variation in $y_{t}$, and is constrained to be linear beyond the end knots so that it can be used for forecasting. In the GDM hazard and GLM models, the effects $\beta_{t,d}$ are defined by a different penalized cubic spline (each with its own smoothness penalty) for each delay index *d*, intended to capture temporal changes in the delay mechanism. In the GDM survivor model, this complexity is substantially reduced a priori by only using one spline $\beta_{t}$ in the model for the expected cumulative proportions $S_{t,d}$. As discussed in Wood (2016), the coefficients of each spline are assigned a multivariate‐normal prior distribution and are penalized to prevent excessive wiggliness through an unknown penalty parameter τ (a scaling factor in the prior precision he matrix). A prior can be put on the more interpretable $\sigma =1/\sqrt{\tau }$, where smaller σ corresponds to higher penalty on wiggliness. The splines are centered to have zero mean, so that fixed effects ι and $\psi_{d}$ are interpretable.

Generally noninformative prior distributions were chosen, detailed in Section 2 of Web Appendix A. All code was written and executed using R (R Core Team, 2019) and all models were implemented using nimble (de Valpine *et al*., 2017), a facility for highly flexible MCMC. The model matrices for the splines were set up using the package jagam (Wood, 2016). Four MCMC chains were run from different initial values and random seeds, until convergence criteria were met (Section 3 of Web Appendix A). The survivor model was computationally fastest (≈30 minute), compared to the hazard (≈60 minute), and GLM (≈120 minute) models. Code and data for reproducing all results are included as the Supporting Information.

### 4.2 Results

Here we discuss ways in which the models differ, while in Section 5.1 of Web Appendix A we discuss the similarity of the temporal and seasonal effects between the models.

We use in‐sample posterior predictive checking (Gelman *et al*., 2014) to check model fit. Replicates of the observed $z_{t,d}$ and of the fully observed $y_{t}$ (weeks 1‐88) are simulated from the respective predictive distributions. We then check whether important statistics of the data are well‐captured by the corresponding predictive distributions.

**Figure 5:**
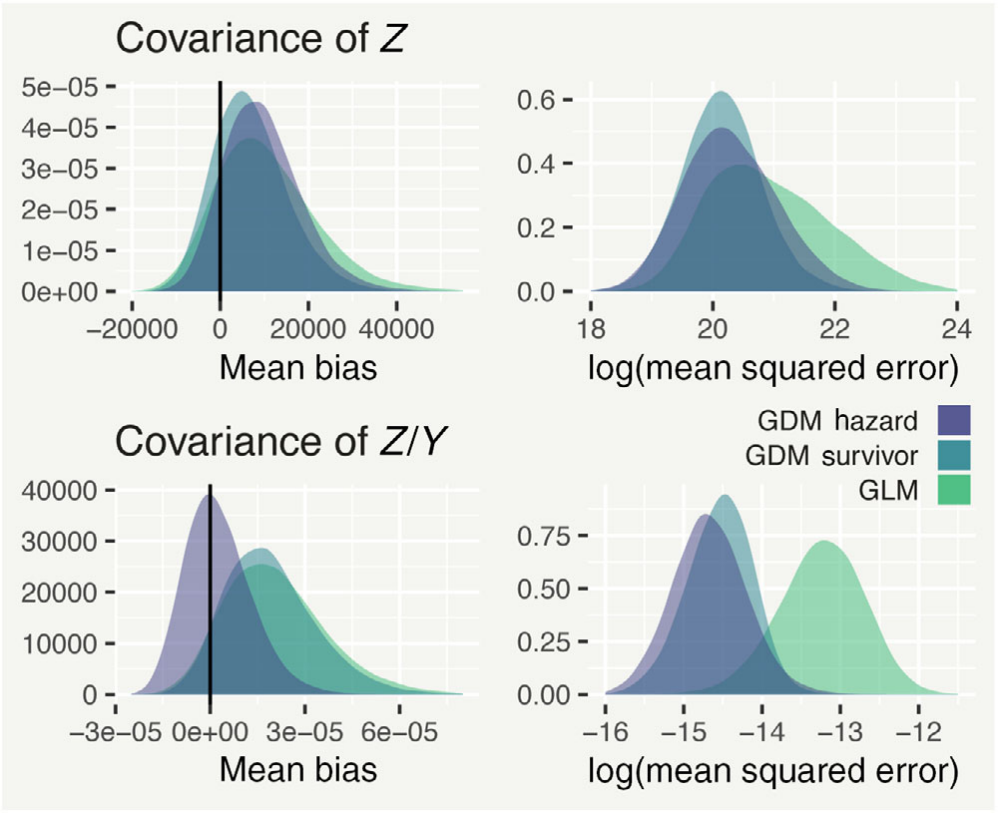
Density plots of the mean bias (left column) and the logarithm of the mean squared error (right column) of the covariance of the partial counts $z_{t,d}$ and the proportion reported in each week $z_{t,d}/y_{t}$. This figure appears in color in the electronic version of this article, and any mention of color refers to that version

We begin by looking at sample estimates of $\text{Cov}[z_{t,d},z_{t,d^{\prime }}]$ and $\text{Cov}[z_{t,d}/y_{t},z_{t,d^{\prime }}/y_{t}]$. The left (right) column of Figure 5 shows the mean difference (mean‐squared difference) between replicated and observed covariances. For $\text{Cov}[z_{t,d},z_{t,d^{\prime }}]$, the survivor model is the least biased and most precise, with the hazard model coming second in precision. For $\text{Cov}[z_{t,d}/y_{t},z_{t,d^{\prime }}/y_{t}]$, the hazard model is the least biased, likely owing to the larger number of parameters compared to the survivor model, while both GDM variants are far more precise than GLM.

Predictive distributions of the sample mean and variance of replicated $y_{t}$ were compared to the corresponding observed statistics in the left and central panels of Figure 7 in Web Appendix A). In both cases, the observed statistic is captured best by the GDM models, though the GLM model also fares relatively well. Additionally, we computed the posterior medians of sorted replicated $y_{t}$, with 95% prediction intervals (shown in the right panel of Figure 7 in Web Appendix A). For all models, the posterior medians match the observed medians closely, indicating the distribution of $y_{t}$ is captured well.

In Section 5.3 of Web Appendix A, we also investigate whether the addition of GD variability leads to tangible improvements over methods relying only on multinomial variability (Section 2.1). In summary, 95% prediction interval coverage for posterior replicates of $z_{t,d}/y_{t}$ is very poor (under 70%) without the GD variability.

**Figure 6:**
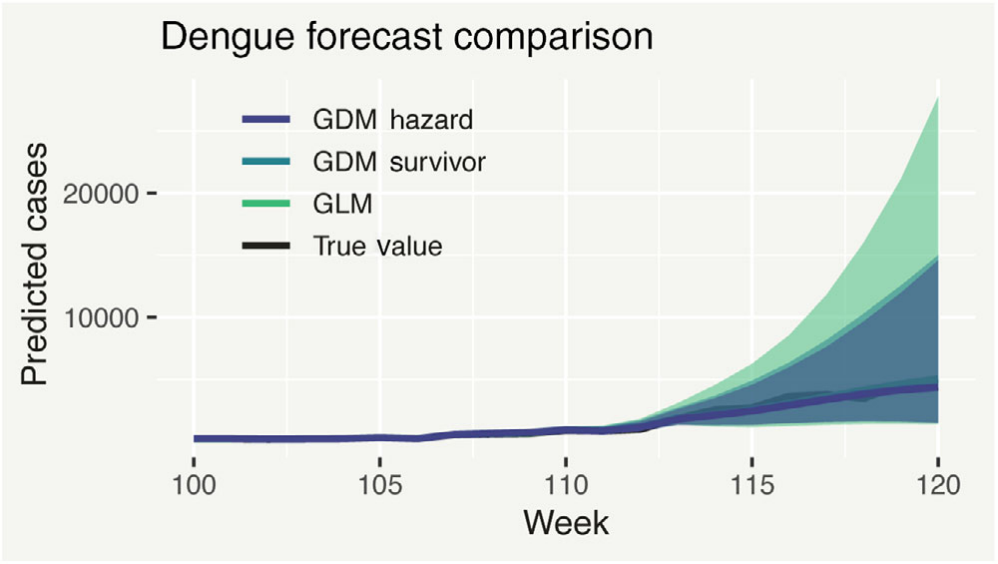
Posterior median predictions of the unobserved/partially observed total dengue cases $y_{t}$, from the GDM hazard, GDM survivor, and GLM models, with associated 95% posterior predictive intervals. This figure appears in color in the electronic version of this article, and any mention of color refers to that version

Finally, we look at nowcasting and forecasting performance. Recall that we are in week $t_{0}=114$ and we wish to predict $y_{t}$ for recent weeks t≤114, as well as forecast the next 6 weeks (t=115,…,120). Figure 6 shows posterior median predicted $y_{t}$ (median and 95% prediction intervals), for *t* = 100, …, 120 (recalling that $y_{t}$ is unobserved for t>88), from the three models. Median predictions from all three models are virtually identical; however in both the nowcasting and forecasting ranges, the two GDM models have far less predictive uncertainty than the GLM. Notably, the survivor model has similar predictive uncertainty to the hazard variant, even though it has much fewer parameters. Importantly, with only 1 week's data (less than 20% of the total as per Figure 2), both GDM models provide a high degree of nowcasting precision, with 95% prediction intervals of approximately 1300‐3500 cases (hazard) and 1300‐3700 cases (survivor) for week t=114. In addition, 80% prediction intervals indicate that within only a few weeks, there is a strong chance (>90%) there will be more than 2000 new cases each week—invaluable information for decision makers.

To further assess the nowcasting and forecasting performance of models based on the GDM framework for these data, as well as to further illustrate such models as a powerful tool for practitioners, we present a more comprehensive prediction experiment in Section 6 of Web Appendix A. In this experiment, we begin with a present‐day week of $t_{0}=100$, making nowcasting predictions for weeks $t_{0}-D+2,\cdots ,t_{0}$ and forecasting predictions for weeks $t=t_{0}+1,\cdots ,t_{0}+4$. We then advance *t*_0_ by 1 week at a time until $t_{0}=140$, covering an entire outbreak cycle (2013), so that we can thoroughly investigate how prediction performance (in terms of precision and reliable quantification of uncertainty) varies with how far the prediction week is from *t*_0_. In summary, we find that both GDM models defined in Section 4.1 display consistently good prediction performance (quantified by prediction interval coverage) for wider intervals (80% and 95%), with disparate performance for narrower ones (50% and 65%). Compellingly, both models performed well across the board when forecasting and nowcasting recent weeks, arguably the most crucial predictions for issuing disease warnings.

## 5 UNDERREPORTING

A related but different challenge is that sometimes, the final observed total count $y_{t}$ is still a (substantial) underestimate of the true count. In disease surveillance, this means cases never being reported, leading to the underestimation of outbreak magnitude. For instance, although reporting of dengue cases to the national surveillance system (SINAN) is mandatory, research suggests the existence of underreporting, owing to issues such as patients not seeking healthcare (Silva *et al*., 2016).

To address this, the GDM framework can be adapted to allow for underreporting. In particular, it can be merged with the hierarchical framework for underreporting presented in Stoner *et al*. (2019a). Suppose that, in addition to the partial counts $z_{t,d}$ and the total counts $y_{t}$, there exist unobserved true counts $x_{t}$, such that $y_{t}\leq x_{t}$. Then the complete modeling framework for delayed reporting and underreporting is given by

(19) $$x_{t}|\lambda_{t},\theta ∼\text{Negative-Binomial}\left(\lambda_{t},\theta \right);$$

(20) $$y_{t}|x_{t},\pi_{t}∼\text{Binomial}\left(\pi_{t},x_{t}\right);\;\log\left(\frac{\pi_{t}}{1-\pi_{t}}\right)=i\left(t\right);\;\;$$

(21) $$z_{t}|y_{t}∼\text{GDM}\left(\nu_{t},\phi_{t},y_{t}\right),$$

where $\lambda_{t}$ is now the incidence rate of the true count $x_{t}$ and $\pi_{t}$ is the reporting rate. Both covariates and random effects can be used to model the reporting rate, represented by the generic function i(t) in (20). Without any observations for $x_{t}$, there is nonidentifiability between a high reporting rate $\pi_{t}$ and a low incidence rate $\lambda_{t}$ or vice versa, but this can be resolved by using at least one informative prior (such as for the overall reporting rate, as discussed in Stoner *et al*., 2019a).

Using this approach means that policy and intervention can be based on predictions for the true number of cases, taking into account both the delayed reporting and underreporting mechanisms to reduce the risk of an undersized response. In contrast, allowing for underreporting in the total count would be much less straightforward using the GLM approach, primarily because the totals $y_{t}$ are not modeled explicitly.

## 6 DISCUSSION

In this article, we have introduced the problem of delayed reporting and its implications. We argued that there are two general approaches to this problem: (a) ones based on a multinomial mixture distribution, with either a time stationary GD distribution or a logistic regression and (b) ones based on conditional independence in the partial counts (GLM). Both approaches are very flexible in terms of incorporating complex temporal structures. However, we argue that they both have limitations: The approaches based on a multinomial mixture are not sufficiently flexible to capture delay mechanisms which are simultaneously heterogeneous in time and overdispersed. The GLM approach, on the other hand, does not explicitly model the total counts. This means it relies on capturing the covariance structure of the partial counts well in order to capture the distribution of the total counts well. This is hindered by the assumption that the partial counts are assumed conditionally independent.

We have proposed a general framework based on a generalized Dirichlet‐multinomial mixture, where the total counts are modeled explicitly and the multinomial probabilities follow a generalized Dirichlet distribution with temporally varying parameters. For this framework, we presented two alternative formulations of the delay mechanism, one which can be considered a natural extension of multinomial logistic regression and another which instead models the expected cumulative proportion of cases reported. Though we present the framework in terms of a general temporal index t∈T, it is also in principle applicable to spatially structured data. Future research is needed to investigate how models for spatial dependence can be incorporated in the models for the total count and the delay mechanism.

We presented a case study of data on reported dengue fever cases in Rio de Janeiro. We used in‐sample predictive model checking to assess the models with respect to how well the distribution of the total number of cases was captured and out‐of‐sample predictive checking to assess performance when nowcasting and forecasting. We found that in every test, models based on the GDM framework had the strongest performance, while the GLM had excessive predictive uncertainty. We also demonstrated in a more comprehensive prediction experiment that the GDM models are both reliable and powerful predictive tools for practitioners.

For these data, it was possible to capture structured temporal variability in the total number of dengue cases simply by combining a seasonal spline and a temporal spline. For data with more complex temporal structures, for example, where disease outbreaks of varying sizes occur at random times throughout the year, a more sophisticated temporal structure may be necessary, which may still be possible within the general model for $\lambda_{t}$ given by (6).

Depending on the experiment, we had 74‐114 weeks of fully observed total counts, plus 26 weeks of partial counts. Predictions were driven by a strong seasonal effect on dengue incidence, which requires at least a year's data to be distinguishable from the temporal effect. Furthermore, we assumed $y_{t}$ is fully reported after 27 weeks, so it is reasonable to consider this the very minimum number of weeks for modeling, with more data desirable. Where the available time series is shorter than the assumed maximum delay *D*, it may be pragmatic to redefine $y_{t}$ as the number of reported cases after a number of weeks $D^{\prime \prime }<D$.

In addition to considering the performance of each model for this particular data set, it is also important to consider other reasons why one might be preferable over the others. The GLM model, for instance, is by far the easiest to implement, especially in a non‐Bayesian setting such as the generalized additive model framework or in an approximate Bayesian setting such as INLA. The GDM framework, however, lends itself more to a full Bayesian treatment, using MCMC. This is because the effects associated with the total count and the effects associated with the delay mechanism are separated into different parts of the model and are related to different parts of the data (the total counts and the partial counts, respectively). In the GLM framework, meanwhile, all of the effects are in the same model and they can end up competing with each other.

In our view, approaches based on the GDM framework are the most interpretable of all of the frameworks discussed here. This is because the delay mechanism, and any associated variability, is completely separated from the process which generates total counts. This in turn makes it easier to adapt the model for a given data set. For example, we can see some evidence in Figure 2 that variability in the relative proportions is higher in some parts of the time series than others. To capture this, it is a fairly trivial modification to model the logarithm of the dispersion parameters $\phi_{t,d}$, as defined in (12), using a penalized spline in time. Knowing that variability in the delay mechanism at a certain time is likely to be lower or higher than usual could further improve nowcasting precision. In the GLM framework, there is no equivalent way of separating temporal structure in the variance of the total counts from structure in the variance of the delay mechanism, as is possible in the GDM framework.

Of the two GDM framework variants we presented, we prefer the survivor as it is more intuitive and easier to simplify. Compellingly, in our case study the survivor model performed as well as the hazard model, despite substantially reduced complexity in the prior model for the delay mechanism. On the other hand, disparate coverage results for narrow prediction intervals in the prediction experiment presented in Section 6 of Web Appendix A suggest care should be taken when specifying the complexity of the delay mechanism.

The GDM framework can also be easily integrated into a hierarchical framework for correcting underreporting, which may be essential in scenarios where the final observed total count is still a substantial underrepresentation of the true count. In such situations, allowing for both the delay mechanism and the underreporting mechanism simultaneously may be crucial for well‐informed decision making.

## Supporting information

Web Appendix A, referenced in Sections 1, 2, 4, and 6, as well as a .zip archive containing all of the necessary code and data to reproduce our results, are available with this paper at the Biometrics website on Wiley Online Library.
