# Supplementary material for: Multivariate hierarchical frameworks for modeling delayed reporting in count data
Source: Biometrics. 2019 Nov 29;76(3):789–98. doi: 10.1111/biom.13188 (PMC7540263; doi:10.1111/biom.13188)
Supplement: Supplementary file 1 — Web Appendix A, referenced in Sections 1, 2, 4, and 6, as well as a .zip archive containing all of the necessary code and data to reproduce our results, are available with this paper at the Biometrics website on Wiley Online Library. [file BIOM-76-789-s001.zip › biom13188-sup-0001-WebAppendix.pdf]

Supporting Information for "Multivariate Hierarchical Frameworks for Modelling Delayed Reporting in Count Data" by

Oliver Stoner and Theo Economou
Department of Mathematics, University of Exeter

Web Appendix A

1 Simulation Experiment

To illustrate the risk posed by not explicitly modelling the total reported counts, as discussed in Section 2 of the main article, we apply four competing models to simulated data and assess their performance. The data was simulated from the following model:

$$y_i \sim \text{Negative-Binomial}(\lambda = 100, \theta = 10), \tag{1}$$

$$\boldsymbol{z}_i \mid \boldsymbol{\pi}_i, y_i \sim \text{Multinomial}(\boldsymbol{\pi}_i, y_i), \tag{2}$$

$$\boldsymbol{\pi_i} \sim \text{Dirichlet}(\boldsymbol{\nu}\phi). \tag{3}$$

In this model, the total counts y_i ($i = 1, ..., n = 100$) arise from a Negative-Binomial model with considerable over-dispersion compared to the Poisson distribution caused by the relatively low value for θ and are each split into three partial counts $z_{i,j}$ ($j \in \{1, 2, 3\}$). These partial accounts arise from a Dirichlet-Multinomial mixture, with mean proportions $\boldsymbol{\nu} = (0.5, 0.2, 0.3)$ and a relatively

low value for the dispersion parameter $\phi = 10$ such that the delay mechanism is also considerably over-dispersed compared to the Multinomial.

1.1 Competing models

Model 1 is a Negative-Binomial model for the total counts with no model for the partial counts $z_{i,j}$. This is the baseline to which we will compare the others.

$$y_i \sim \text{Negative-Binomial}(\lambda, \theta). \tag{4}$$

Model 2 is a marginal Negative-Binomial model for the partial counts $z_{t,d}$, which ignores both the dependence between the $\boldsymbol{z}_t$ and the over-dispersion of the delay mechanism. This approach is conceptually similar to the one proposed by Salmon et al. (2015).

$$z_{i,j} \sim \text{Negative-Binomial}(\nu_j \lambda, \theta). \tag{5}$$

Model 3 extends Model 2 by incorporating a Dirichlet model for the Multinomial proportions $\boldsymbol{\pi}_i$. The Dirichlet can capture the over-dispersion of the delay mechanism, such that it does not have to be absorbed by the Negative-Binomial dispersion parameter θ.

$$z_{i,j} \mid \pi_{i,j} \sim \text{Negative-Binomial}(\pi_{i,j}\lambda, \theta), \tag{6}$$

$$\boldsymbol{\pi}_i \sim \text{Dirichlet}(\boldsymbol{\nu}\phi). \tag{7}$$

Finally, **Model 4** is the marginal model for the partial counts $z_{t,d}$ which both accounts for the dependence between the $\boldsymbol{z}_t$, using the Poisson-Gamma mixture characterisation described in (16)-(18), and accounts for over-dispersion with a Dirichlet model for $\boldsymbol{\pi}_i$.

$$z_{i,j} \mid \pi_{i,j}, \gamma_i \sim \text{Poisson}(\pi_{i,j}\gamma_i), \tag{8}$$

$$\boldsymbol{\pi}_i \sim \text{Dirichlet}(\boldsymbol{\nu}\phi), \tag{9}$$

$$\gamma_i \sim \text{Gamma}(\theta, \lambda\theta^{-1}). \tag{10}$$

For all parameters, non-informative Exponential prior distributions with mean 10000 were specified. The models were implemented using NIMBLE and four chains were run for a total 20k iterations, discarding 10k as burn in. To give all models the best chance of capturing the correct parameter values, all chains were initialised at the true values. Convergence was assessed by computing the multivariate potential scale reduction factor (described in more detail in Appendix 3) for all parameters and obtaining a value of less than 1.05 for each model.

1.2 Results

Figure 1 shows the posterior distributions for λ, θ and, where applicable ϕ, from each model. The dotted line shows the baseline Model 1.

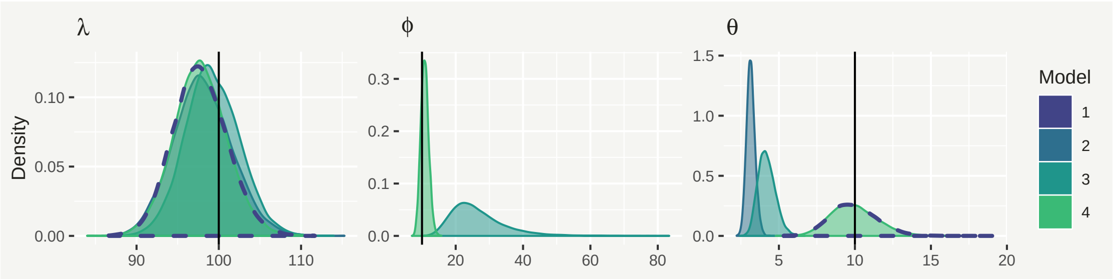

Figure 1: Posterior density plots of parameters λ (left), ϕ (centre) and θ (right), from each model.

Whilst all models were able to correctly capture the true value of λ (though Models 1 and 4 were more certain than Models 2 and 3), only Models 1 (baseline) and 4 were able to correctly capture the Negative-Binomial dispersion parameter θ. In the case of Model 2, this is likely because the lack of an over-dispersion model in the delay mechanism meant θ had to absorb the additional variability. Model 3 was closer to the true value of θ but still very far off (despite its Dirichlet model for over-dispersion). The consequence, as shown in Figure 2 is that both Models 2 and 3 grossly over-estimate the variance of the total counts y_t, when simulating

posterior replicates.

In contrast, by both allowing for an over-dispersed delay mechanism and correctly incorporating the positive covariance in the partial counts $\boldsymbol{z}_t$, Model 4 is able to match the baseline model in capturing θ correctly and, as a result, the variance of y_t.

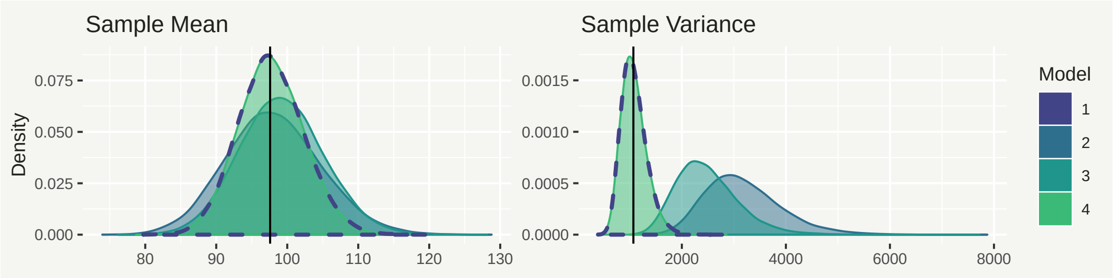

Figure 2: Density plots of the sample mean (left) and variance (right) of posterior replicates of total counts y_i, from each model. The dotted line shows the baseline Model 1.

The conclusion we draw from this experiment is that, in a situation where the Multinomial model for the partial (delayed) counts $z_{t,d}$ is over-dispersed, failing to take into account this over-dispersion and/or ignoring the dependency structure of the $\boldsymbol{z}_t$ can translate to substantial over-estimation of the variance of the total counts y_t.

2 Prior Distributions

For the dengue data case study in Section 4, the Negative-Binomial dispersion parameters (θ) and GDM dispersion parameters ϕ_d were assigned relatively non-informative Exponential(0.01) prior distributions. In the latter case most of the prior density is over values of ϕ_d which result in a modest contribution from the Generalized-Dirichlet component to the overall variance of the GDM, without ruling out higher values which correspond to a Multinomial situation. Relatively

non-informative Normal$(0, 10^2)$ prior distributions were specified for the global intercept parameter ι and also for the delay-specific intercept parameters ψ_d. In the GDM Hazard model, the intercept parameters ψ_d represent the means of relative proportions at the logistic level. For these parameters we specified Normal prior distributions with the means chosen so that the prior mode implies approximately equal amount of cases being reported in each week of delay, with the variance chosen so that they are relatively non-informative. In the GDM Survivor model, the ψ_d are intended to capture the curve seen in the right plot of Figure 3, with the spline β_t allowing this to shift up and down over time. As we are modelling the cumulative proportion, an additional constraint that $\psi_d > \psi_{d-1}$ was imposed.

We specified Half-Normal$(0, 1)$ prior distributions for the penalty parameters for splines α_t and η_t. This imposes a relatively strong smoothness penalty on the effects α_t and η_t, which are supposed to capture medium-to-long term trends in the incidence of dengue cases. We relaxed this penalty slightly for the effects $\beta_{t,d}$ (β_t in the GDM Survivor model) by specifying weaker Half-Normal$(0, \sqrt{2})$ priors.

3 Convergence of MCMC Chains

For each model of the dengue data, convergence of the four chains was assessed by visual inspection of trace plots and by computing the Multivariate Potential Scale Reduction Factor (MPSRF) (Brooks and Gelman, 1998) of a selection of model parameters. This compares the variance between the chains to the variance within the chains. If the two variances are similar then this typically results in an MPSRF of less than 1.05. Starting from different initial values and obtaining an MPSRF of around 1.05 or less gives the best indication that the chains have converged to the posterior distribution.

- For the GDM Hazard model, we computed the MPSRF of every 10th λ_t

$(\lambda_{10}, \lambda_{20}, ...)$, θ, every 10th $\beta_{t,d}$ and the ϕ_d. The model was run for a total of 400k iterations, discarding the first 200k as burn-in and thinning by 20 to save memory. The MPSRF was computed to be 1.05 indicating that the model had converged.

- For the GDM Survivor model, we computed the MPSRF of every 10th λ_t $(\lambda_{10}, \lambda_{20}, ...)$, θ, the ψ_d, every 10th $\beta_{t,d}$ and the ϕ_d. The model was run for a total of 200k iterations, discarding the first 100k as burn-in and thinning by 20 to save memory. The MPSRF was computed to be 1.01, indicating that the model had converged.

- For the GLM model, we computed the MPSRF of every 10th $\mu_{t,d}$ and θ. The model was run for a total of 800k iterations, discarding the first 400k as burn-in and thinning by 40 to save memory. The MPSRF was computed to be 1.04.

4 Sensitivity to the Number of Delays Modelled

As discussed in Section 4.1 of the main article, instead of modelling the partial counts $z_{t,d}$ for all $d = 1, \ldots, D$, we can instead only model explicitly counts up to $d = D'$, with the remainder r_t modelled either implicitly (in the GDM framework) or explicitly as a single aggregated count (in the GLM framework). Here we investigate the sensitivity of predictions to the choice of D', in our application to the dengue data with present day $t_0 = 114$.

We fitted the GDM Survivor model from Section 4.1 of the main article to the dengue data, starting with $D' = 3$ and ending with $D' = 11$. First we note that there is a computational cost for each additional delay week we model explicitly. Figure 3 shows run-times for four MCMC chains and 200k iterations. We can see that the overall computation time appears to increase linearly with D'.

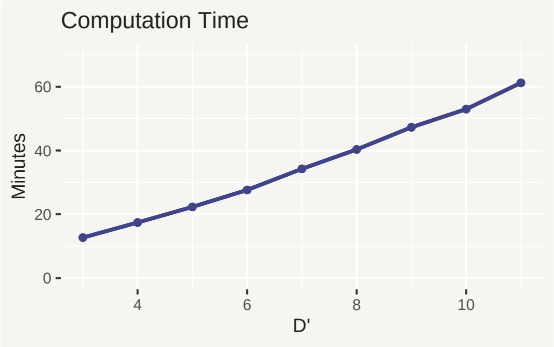

Figure 3: Computation times for runs of the GDM Survivor model, with 200k MCMC iterations and four chains, for varying D' (the number of delays modelled explicitly).

To illustrate how predictive performance varies with D', Figure 4 shows predicted (median, 50% and 97.5% intervals) total dengue cases y_t for weeks $t = 98$, 102, 106, 110, 114 and 120. Looking at the two bottom-right plots, we can see that both the now-casts for the present week (114) and forecasts for week 120 are seemingly invariant in D'. In predictions of y_t for previous weeks (which we still haven't fully observed), lower values of D' do lead to increased predictive uncertainty. However, the value of D' below which uncertainty begins to increase, is higher for earlier weeks (e.g. week 98) than more recent ones (e.g. 110).

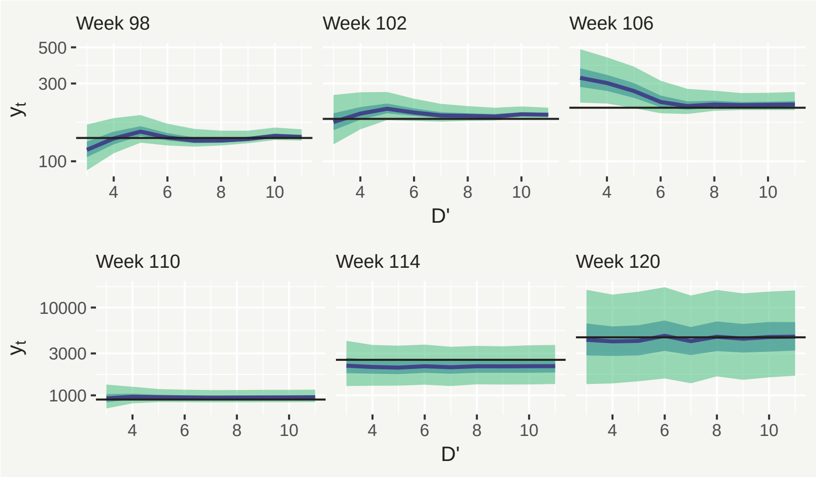

Figure 4: Predictions (median, 50% and 97.5% prediction intervals) of the total reported dengue cases y_t, for weeks $t = 98$, 102, 106, 110, 114, 120.

To understand why this might be, consider that we only have one partial count ($z_{t,1}$) for the present week (114). This means that, regardless of our choice of D', we still only have one partial count for that week to inform our predictions. In

contrast, for week 106 we have 9 partial counts for that week, so values of D' less than 9 means ignoring information in at least one count, leading to increased predictive uncertainty. Moreover, the lower the value of D', the more counts are ignored. It is worth noting, though, that the choice of D' also affects the number of counts available for previous weeks. For example, if $D' = 2$ then, although we still only have one partial count for the present week, we have two counts to inform predictions for the previous week, which may in turn lead to more precise prediction for the present week compared to $D' = 1$. This effect does appear to be negligible for this data, however. Our main conclusion from this experiment is, therefore, that the choice of D' can be viewed as a trade-off between computation time (which varies linearly with D') and the number of time steps t into the past for which predictions are required to be as precise as possible. For example, in the case where a practitioner is only interested in now-casting y_t for the present week and in forecasting, then it may be feasible to only model the first partial count ($z_{t,1}$) explicitly. In other applications, e.g. for predicting long-term insurance losses, or if the y_t represent disease counts at a finer temporal resolution, a larger value of D' is more sensible.

5 Further Analyses

In this section we present some additional analyses from the application of competing models to the dengue fever data.

5.1 Temporal and seasonal effects

In the main article, the differences in the results between models were presented. Here we present aspects of the results that were similar. Figure 5 shows the posterior mean of α_t and (η_t) from all three models. Both effects (temporal and

seasonal) are very similar in shape across models: the left panel indicates a persistent increase of dengue incidence in 2012, which makes sense given the more severe outbreak shown in Figure 4 of the main article, while the right panel shows a strong seasonal effect, with a much higher incidence rate in the first half of the year than the second.

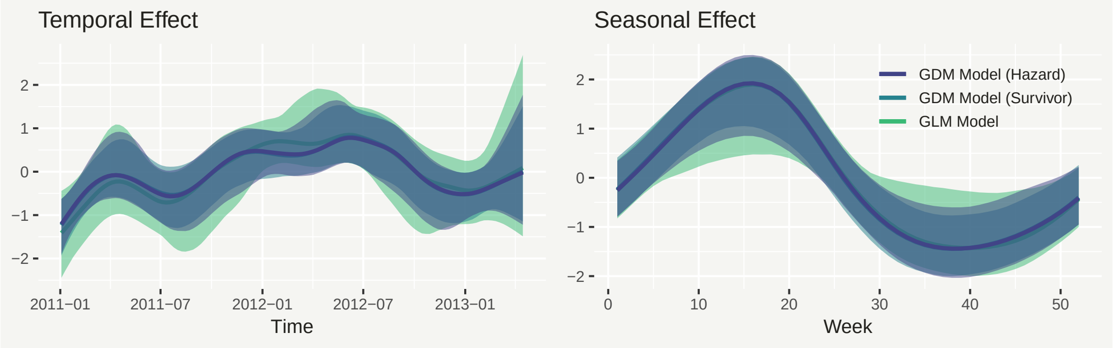

Figure 5: Posterior median temporal (α_t) and seasonal (η_t) effects on the log incidence rate, from the GDM Hazard, Survivor and GLM models, with associated 95% credible intervals.

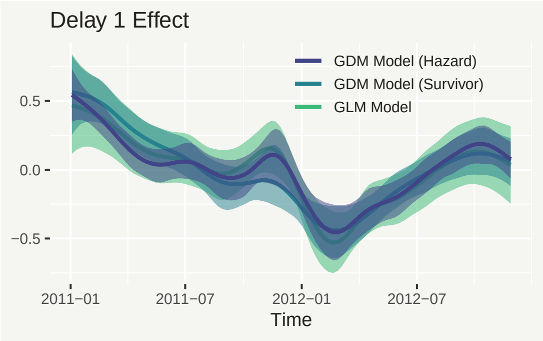

Figure 6: Posterior median (scaled to have range 1) delay spline effect corresponding to counts reported in the same week they occurred $z_{t,1}$ in the GDM Hazard and GLM modes ($\beta_{t,1}$), and the delay spline effect β_t in the GDM Survivor model, with associated 95% credible intervals.

Similarly, Figure 6 shows that—although not strictly comparable due to different link functions (logistic for GDM Hazard, probit for GDM Survivor and log for GLM)—the temporal effects (scaled to have range 1) corresponding to the number of cases reported in the same week they occurred (d=1) are very similar between

the three models. For example, all models show a distinct drop in proportion of cases reported in the week they occurred during the 2012 outbreak.

5.2 Additional plots

Here (Figure 7) we present some posterior predictive density plots which are referenced in the main article.

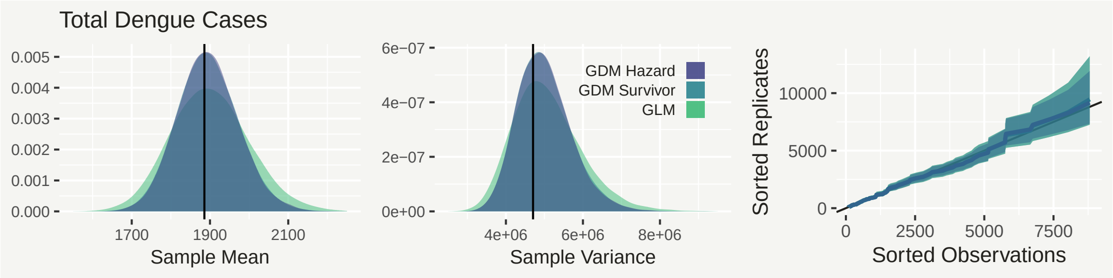

Figure 7: Density plots of the sample mean and sample variance (left and central panes) of the posterior replicates of the fully observed (weeks 1-104) total dengue cases (y_t) from the GDM Hazard, GDM survivor and GLM models. The vertical lines represent the corresponding statistics from the observed data. The right panel shows the mean of the sorted replicates of the total dengue cases y_t, with associated 95% posterior predictive intervals.

5.3 Comparison with Multinomial

While the Generalized-Dirichlet component in the GDM framework affords extra flexibility, it also makes the model more complex. For this reason, it's important to assess whether this aids substantially in fitting the dengue data well. This can be done by simulating posterior replicates of the proportion reported in each week of delay ($z_{t,d}/y_t$) from the GDM and again from the same model but in the limiting case when $\phi_d \to \infty$, such that $z_{t,d}|y_t$ is Multinomial. We simulate from the GDM Hazard framework as it is a direct extension of the Multinomial

regression approach. For example, we computed the coverage of the 95% posterior predictive intervals for the proportions $z_{t,d}/y_t$ reported in the same week they occurred ($d = 1$) and in the second week ($d = 2$), for both the replicates with GD variance and without. These intervals are plotted over the time period in Figure 4 of Web Appendix A. Without the GD variance, an excessively high number of observations are not captured: the prediction interval coverages with the GD variance were just over 95%, indicating a good fit to this data. In contrast, less than two-thirds of observations are covered without the GD variance.

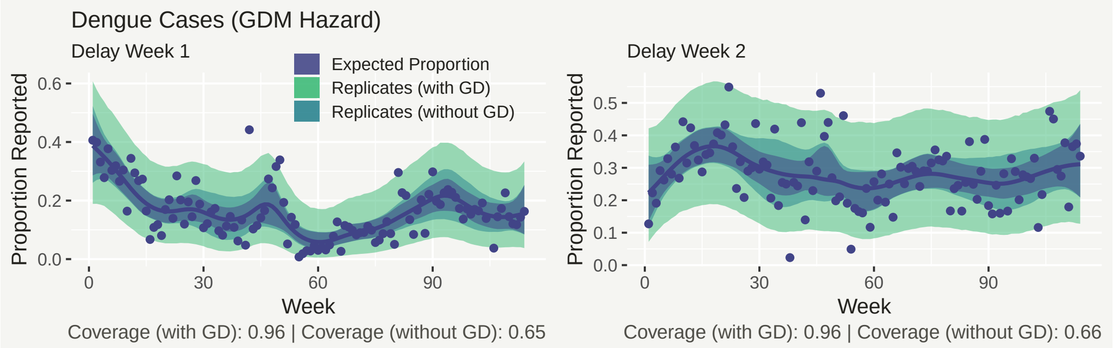

Figure 8: Posterior median proportion, from the GDM model, of dengue cases reported in the first (left) and second (right) weeks, with associated 95% prediction intervals.

6 Prediction Experiment

For now-casting and forecasting applications, the most important characteristic of any model is arguably the quality of the predictions for the partially observed or wholly unobserved total counts y_t. To compare models in Section 4 of the main article, we applied them to the dengue data in the scenario where we find ourselves wanting to make predictions at present-day week $t_0 = 114$. To more comprehensively assess the quality of now-casting and forecasting predictions, as

well as to mimic the real world usage of these models for issuing disease warnings, we consider a more extensive experiment where we advance t_0 in a week-by-week rolling scenario, from $t_0 = 100$ all the way up to $t_0 = 140$ (covering more than 9 months).

6.1 Methodology

Let δ denote the difference between the present-day t_0 and the week we are making predictions for. For example, at present-day week $t_0 = 100$, the next week (101) corresponds to $\delta = 1$ and the previous week (99) corresponds to $\delta = -1$. For each present-day t_0, we have completely observed total counts y_t for $t = 1, \ldots, t_0 - D_{max} + 1$, partially observed counts for $t_0 - D_{max} + 2, \ldots, t_0$, and no observations for weeks $t > t_0$. We can then fit a model to this data and predict (now-cast) the partially observed y_t ($\delta = -D_{max} + 2, \ldots, 0$) and forecast the completely unobserved y_t for subsequent weeks, which we take to be the next four weeks ($\delta = 1, \ldots, 4$).

Starting from a present-day week of $t_0 = 100$, we then advance one week so that present-day is week $t_0 = 101$ and repeat the prediction. If we do this many times, in this case for weeks $t_0 = 100, \ldots, 140$, we can investigate the quality of predictions for y_t and how this varies with δ. For example, we might expect our prediction for week $\delta = 0$ (present-day) to be more precise than our prediction for week $\delta = 4$ (four weeks ahead), though we would hope that the prediction interval coverage is similar for both. We choose this range of weeks because it completely covers a whole outbreak cycle (2013).

6.2 Results

Figure 9 shows sets of now-casting and forecasting predictions (medians and 95% prediction intervals) of the total reported counts y_t for GDM Survivor models

(as defined in Section 4.1 of the main article), fitted to present-day weeks $t_0 = 100, 110, 120, 130, 140$. The plot shows how the now-casting/forecasting evolves as time advances: initially at $t_0 = 100$, we are following several months of low dengue incidence and the forecasts correctly show no sign of any epidemic on the horizon. At time $t_0 = 110$, the now-casting predictions correctly predict we are in the early stages of an epidemic and the forecasts suggest this is very likely to worsen in subsequent weeks. At time $t_0 = 120$ we are at the peak of the epidemic. While uncertain, the forecasts suggest that the number of new dengue cases will decrease in each of the four subsequent weeks, a change which is likely driven by the seasonal effect. Also plotted are the 95% prediction interval coverages for each set of now-casting and forecasting predictions. Notably, they are all around 95%, suggesting they are quantifying the predictive uncertainty well.

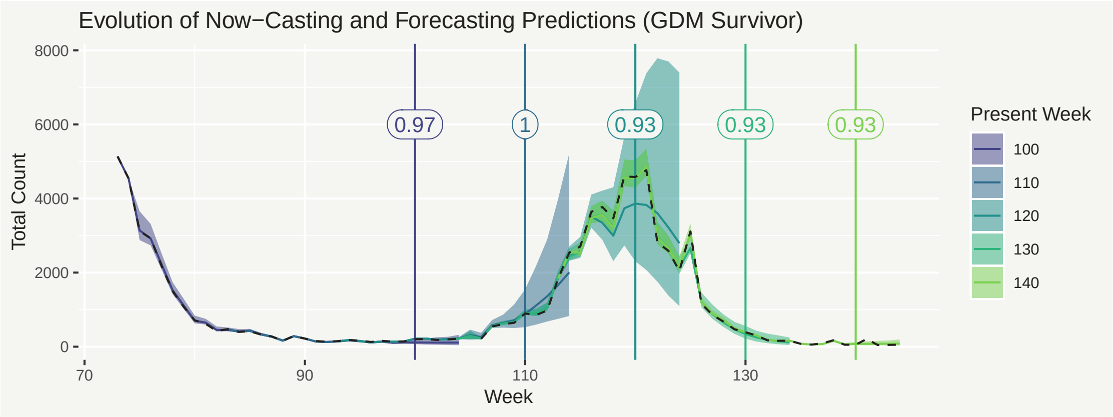

Figure 9: Median predicted total reported dengue cases (y_t) for models fitted with present weeks $t = 100, 110, 120, 130, 140$, with 95% prediction intervals and associated coverage values.

As expected, the further into the future the forecasts are (or the closer to the present the now-casts are), the greater the prediction uncertainty. Figure 10 illustrates this in more detail, with scatter plots comparing the true totals y_t to the corresponding median predictions (and 95% predictions intervals) for each

$\delta = -5, \ldots, 4$. For example, for every model run with a different present-day week in $t_0 = 100, \ldots, 140$ we have a now-casting prediction for that week ($\delta = 0$). These are illustrated in the bottom-left panel of Figure 10. Similarly, for each t_0 we have a prediction for four weeks into the future ($\delta = 4$), shown in the bottom-right panel. Looking from left to right, first across the top row and then across the bottom row, we can see that as δ increases, the differences between the predictions and the true values increases (as well as the predictive uncertainty, as quantified by 95% intervals). This makes sense, as for each consecutive decrease in δ, we have an additional observed partial count $z_{t,d}$ to inform our prediction of y_t. Once again we plot 95% prediction interval coverages, but this time these are for the sets of predictions $t_0 = 100, \ldots, 140$ corresponding to each value of δ. Importantly, these are all quite high, indicating that decent 95% prediction interval coverage is achievable for both now-casting and forecasting.

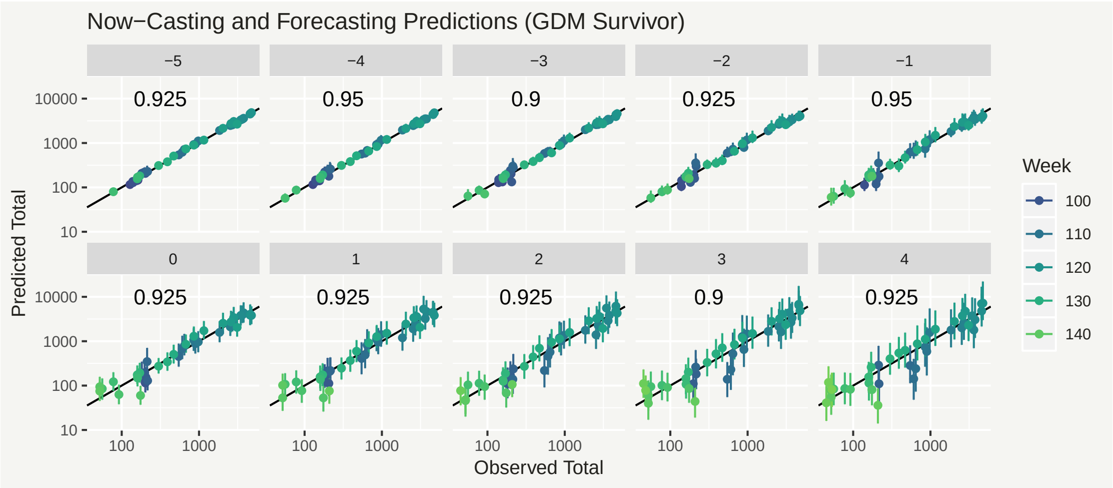

Figure 10: Predicted (median and 95% intervals) total counts y_t, with each panel corresponding to a different value of δ (the difference between the present-day t_0 and the prediction week).

To see whether prediction interval coverage changes systematically as δ increases or decreases, Figure 11 shows the 50%, 65%, 80% and 95% prediction

interval coverages for each $\delta = -25, \ldots, 4$. This time we also include predictions from the GDM Hazard model. We superimpose LOESS smoothing estimates to aid in spotting systematic trends. Looking first at the 50% and 65% prediction intervals from the Survivor models, we can see that for most of the range of δ, the coverage is substantially lower than the desired values of 50% and 65%, respectively. The implication of this is that, for this dataset, 50% and 65% P.I.s are probably too narrow. The 80% and 95% P.I. coverages, meanwhile are very close to the desired values of 80% and 95%, respectively.

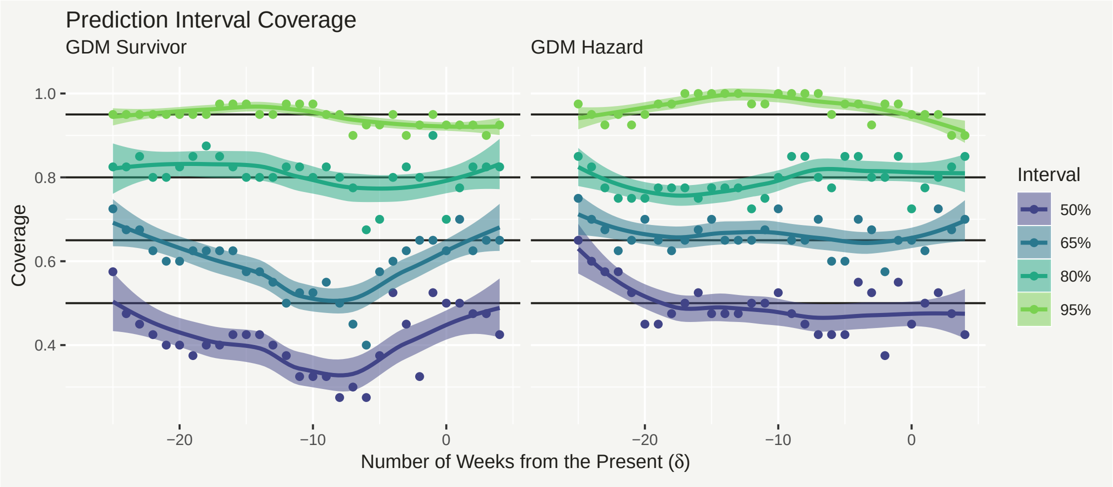

Figure 11: Prediction interval coverages (50%, 65%, 80% and 95%) for varying values of δ, the difference between present-day (t_0) and the prediction week.

The coverage values for the Hazard models are broadly more consistent across the range of δ. Crucially, the prediction interval coverages from both models are very similar and close to their desired values when now-casting recent weeks (e.g. $\delta = -2, -1, 0$) and when forecasting ($\delta = 1, 2, 3, 4$); arguably the most important predictions in terms of issuing warnings and reacting to outbreaks.

While it is important that the prediction interval coverages are not too much lower than their claimed values, for now-casting and forecasting applications it is equally important that they are not excessively high. This would correspond

to excessive uncertainty, which makes the predictions less useful to practitioners. Compellingly, the prediction intervals don't tend to exhibit excessive coverage for either model.

6.3 Conclusion

Recall that the difference between the two variants of the GDM framework is how the expected delay structures are characterised, with everything else the same. For this particular dataset, we specified a simpler (a-priori) characterisation of the expected delay mechanism for the Survivor model, with only one delay spline compared to 8 in the Hazard model. Clearly, the prior complexity of the expected delay model makes a difference in terms of prediction interval coverage, with the simpler model (Survivor) more likely to have too low coverage (e.g. the 50% intervals in Figure 11) and the more complex model more likely to have too high coverage (e.g. the 95% prediction intervals in Figure 11). As this is virtually the only difference between the two models, this experiment suggests care should be taken in designing the complexity of the expected delay mechanism, for example by conducting a similar experiment to this one.

That said, the main lesson from this experiment is that both GDM models are extremely powerful tools for now-casting and forecasting. We have shown that a practitioner using either model every week over the course of the 2013 outbreak would have had access to good-quality predictions for the total reported dengue cases y_t, particularly when now-casting recent weeks and when forecasting where the 50%, 65% and 80% and 95% intervals all show near-ideal coverages.

References

Brooks, S. P. and A. Gelman (1998). General methods for monitoring convergence of iterative simulations. Journal of Computational and Graphical Statistics 7(4), 434–455.

Salmon, M., D. Schumacher, K. Stark, and M. Höhle (2015). Bayesian outbreak detection in the presence of reporting delays. Biometrical Journal 57(6), 1051–1067.
